# A Fully-Integrated Memristor Chip for Edge Learning

**DOI:** 10.1007/s40820-024-01368-7

**Published:** 2024-04-02

**Authors:** Yanhong Zhang, Liang Chu, Wenjun Li

**Affiliations:** School of Electronics and Information, Hangzhou Dianzi University, Hangzhou, 310018 People’s Republic of China

**Keywords:** Computing in memory, Edge learning, Fully-integrated chip

## Abstract

- The fully-integrated memristor chip for edge learning provides a solid foundation for neural network computation.
- The fully-integrated memristor chip enables efficient object recognition in noisy backgrounds while minimizing energy consumption.
- The computing-in-memory chip represents an innovative and interdisciplinary technology that extends beyond multiple research domains.

It is still challenging to fully integrate computing in memory chip as edge learning devices. In recent work published on *Science*, a fully-integrated chip based on neuromorphic memristors was developed for edge learning as artificial neural networks with functionality of synapses, dendrites, and somas. A crossbar-array memristor chip facilitated edge learning including hardware realization, learning algorithm, and cycle-parallel sign- and threshold-based learning (STELLAR) scheme. The motion control and demonstration platforms were executed to improve the edge learning ability for adapting to new scenarios.

With the rapid development of artificial intelligence (AI), the data storage and computation in neural networks are urgent to overcome the challenge of von Neumann computing architecture. In traditional computing architecture, the data storage and computation executed in separated units cause low computing speed, high energy consumption and large-scale stacking, which hamper the processing capability for edge learning devices in complex scenarios. Thus, it is desirable to develop new-principal computing devices. The human brain has a neural network circuit composed of ~ 10^11^ neurons and ~ 10^15^ synapses connected each other. With the distributed and parallel computing, the human brain has powerful memory and high computing speed. Inspired by the human brain, artificial synapses can realize neuromorphic computing to be the most potential channel for breaking the computing bottlenecks.

Memristor has been regarded as a promising alternative for neuromorphic computing through simulating biological synapse functions. In memristor device, the nonlinear resistance is determined by the excitation history and changes continuously to indicate the memory functions. Importantly, the memristor can simulate the operation mechanism of human brain neurons to perform matrix multiplication and addition operation through integrating the intrinsic storage and computing. Therefore, the memristor is provided with transcendent neuromorphic processor of saving data movement overhead to greatly improve computing speed, save processing energy and reduce stacking scale.

For practical applications, memristors are still hindered by the following obstacles. The ion migration is the commonest working principle of memristors; however, the ion discreteness and instability severely reduce the computational accuracy. Besides, the integration of neuromorphic memristor chip requires high level of coordination to achieve super potent data processing and computing. Moreover, the integration of memristor arrays must address the IR drop. Furthermore, neuromorphic memristor chip necessitates architecture and algorithm design for efficient operation and intelligent functionality. Lastly, lack of electronic design automation (EDA) tool-chains and compilers impede the computing. Significantly, a recent work faced the challenges to address the above obstacles through a fully-integrated chip based on neuromorphic memristors for edge learning.

## Memristors

In conventional memristors, the conductive filaments (CFs) are usually formed without regular structures. Moreover, the random migration of ions and relaxation effect of ion distribution cause the discreteness and instability in resistive-switching (RS) characteristics [5]. To overcome the limitations, a sandwiched “TiN/HfO_x_/TaO_y_/TiN” memristor was developed, where the TaO_y_ capping is an enhanced thermal layer to improve the homogeneity and stability through controlling the electric field and temperature distribution within the HfO_x_ layer [2]. In the memristor, the CFs consist of stable distribution and dynamic part. The stable distribution forms a strong connection with the bottom electrode. By applying a soft but sufficiently strong RESET pulse, the dynamic part can be erased without affecting the stable distribution. This alternated operation of SET and RESET is performed during learning iterations to ensure efficient recovery and establish similarity or correlation between RESET and initial conductance values [1]. The delicate balance between SET and RESET maintains the correlated filamentary switching phenomenon. The memristor has a high yield for 5-bit (32-level) programming, along with a minimum success rate of 99.69% across all the programmed conductance states, while ensuring low operating energy and long-term retention [4].

## Neuromorphic System

**Fig. 1:**
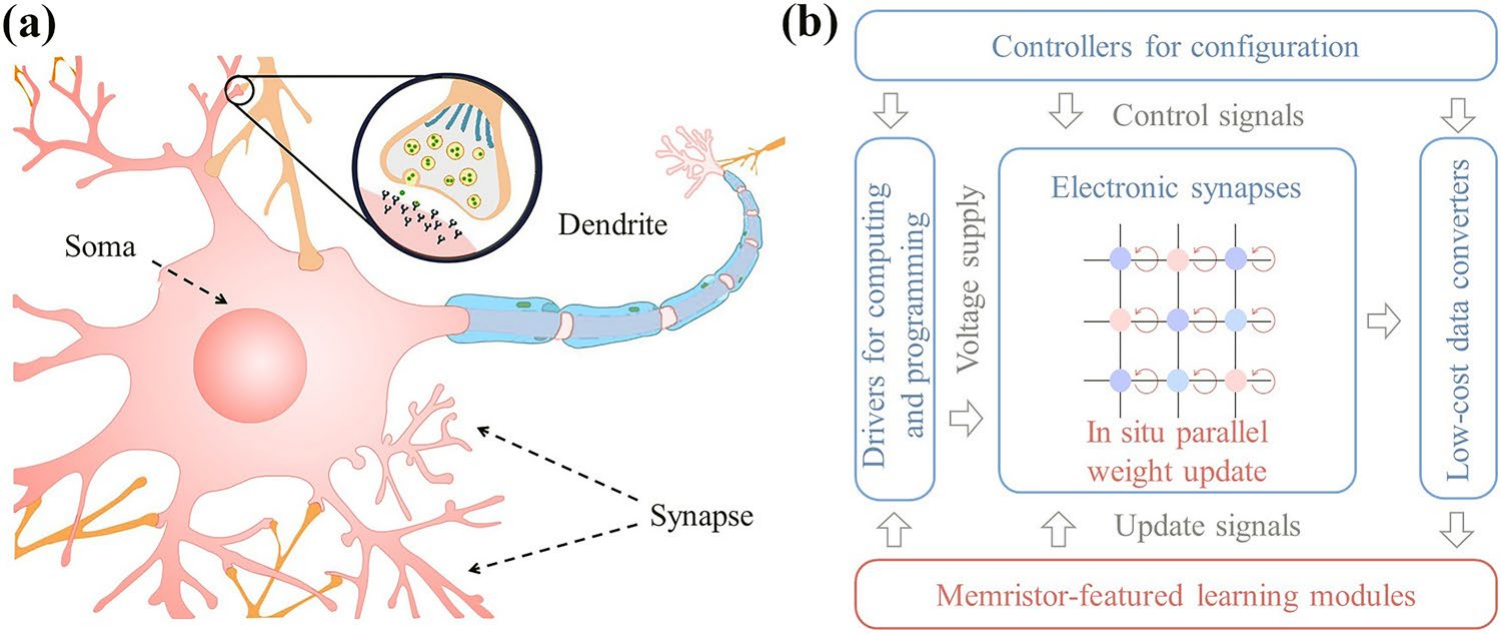
**a** Dendrites connect the soma and synapses. **b** Architecture design of the fully integrated memristor chip [1]

In nervous system, a neuron is consisted of dendrites, one soma, and axons, which is capable of unequally processing thousands of synaptic inputs. Within a defined time interval, postsynaptic potentials from same and different synapses are nonlinearly integrated to encode both space and temporal information in the dendrites. The soma further integrates the potentials from the dendrites and determines whether to generate spikes based on the integrated amplitude within the defined time window. The electrical conductance along with resistance change is from the ion migration in memristors, resembling the phenomena in biological synapses and neurons. The fundamental computational characteristics of neurons can be understood deeply, where the synapses represent plastic weights, and the soma provides integration and spike-firing functions (Fig. 1a). Nonlinear integration and filtering of postsynaptic potentials enable the network to process hierarchical information on different dendritic branches.

In memristor, when an applied voltage is below the threshold, the device remains OFF state, without current responses for pulses. In contrast, voltage pulses over the threshold turn on the device states, and continuously increase the current responses with prolonging time [3]. The incorporation of dendritic functions is crucial for implementing an artificial neural network that efficiently recognizes objects under noisy backgrounds. A single memristor can simulate voltage-gated NMDA channels for dendritic functionalities. After introducing dendrites, the recognition accuracy of SVHN dataset significantly improves, and dynamic energy consumption reduces by 30 times.

## Memristor Array

The crossbar array as a highly compacted arrangement, is frequently employed in memristor integration. In intersection point, the current is determined by multiplying the input voltage and conductance according to Ohm's law. And the total current of each column can be calculated by summing the currents of corresponding intersection points based on Kirchhoff's current law. During the computing process, the intermediate data can be stored directly in the form of conductance and then organized into large arrays. This enables the implementation of vector–matrix multiplication, which is the fundamental computation in deep learning algorithms. The analog characteristic of crossbar array facilitates the efficient data execution, while the traditional digital computing often struggles with the resource-intensive nature. Furthermore, direct analog computation eliminates the analog-to-digital (ADC) or digital-to-analog (DAC) conversion, which saves a massive of energy [5].

In conventional crossbar arrays of one-transistor-one-resistor (1T1R) configuration, the two memristor cells within differential pair are connected to separate source lines, where the subtraction is performed with digital way. The IR drop is accompanied by the flow of an electric current within the internal circuitry, which reduces the total performance, increases the energy consumption and damages the integrity of signals. While in crossbar arrays of two-transistor-two-resistor (2T2R) configuration, the two memristor cells within a differential pair are connected to the same source line, which allows for direct subtraction in the current domain. This 2T2R design can significantly reduce the source-line current and address the IR-drop issue.

## Integrated Memristor Chip

A memristor-featured STELLAR architecture has been proposed, which provides neuro-inspired computing capability for fully on-chip learning (Fig. 1b). The proposed scheme saves excessive write-and-read costs in conductance tuning operations, compared with the write verification scheme in the BP algorithm. In the STELLAR architecture, the weight update calculation is accomplished through custom-designed circuits, and the conductance tuning operation is finished without verification process, indicating the capability to accommodate tuning issue such as nonlinearity and asymmetry, for software comparable accuracy. Moreover, on-chip calculation module was specifically designed to determine weight update directions using only the signs of inputs, outputs, and errors, eliminating the need for high-precision formats. This streamlined design approach alleviates the burden on circuit design and avoids unnecessary overhead during on-chip learning. Furthermore, a cycle-parallel conductance tuning scheme was introduced, enabling conductance tuning to be performed in a row-by-row parallel fashion. This innovative scheme significantly reduces energy consumption, and effectively addresses the limited durability of memristors [1].

The system consists of two crossbar memristor arrays, where the larger one with 1568 × 100 memristors has 2T2R configuration. These memristors collectively represent a weight matrix of 784 × 100, which is essential for neural network models. The weight values undergo offline training using an off-chip training approach and are subsequently loaded onto the crossbar arrays for parallel matrix multiplication. This demonstrates the remarkable attributes of "storage and computation integration" with numerous nodes. Furthermore, a smaller 1T1R array consisting of 100 × 20 memristors was incorporated, representing weight matrix of 100 × 10. This arrangement enables on-chip learning capability with weight update logic. The two crossbar arrays combine to form a three-layer 784 × 100 × 10 neural network structure, which is suitable for performing small-scale artificial intelligence algorithm tasks. The 28 nm fabrication process of chip provides the excellent integration density and performance. This process facilitates the implementation of a greater number of functional units on chip, which is pivotal in the design of computing in memory chip.

## Algorithm

A hybrid training framework consisting of on-chip improvement training and off-chip training was used to improve the accuracy of algorithm. The on-chip learning of memristor chip involves three stages including Forward (FWD), SET, and RESET. In FWD stage, vector–matrix multiplication (VMM) operation is performed using memristor arrays and on-chip ADCs [1]. The SET and RESET stages involve determining weight update direction and conducting conductance tuning operation. The cycle-parallel STELLAR scheme takes advantage of the bidirectional analog switching characteristic of memristors, saving pulses by updating only one memristor in the 2T2R weight unit. In off-chip training, each synaptic weight is represented by a pair of memristor cells. The positive and negative cells are mapped to adjacent columns in a 1T1R array. During weight transferring, the negative cell is programmed to the lowest conductance state if the synaptic weight is positive, and vice versa.

The computing-in-memory chip demonstrated its ability to enhance learning with new samples. In the motion control task of a light-chasing car, a control algorithm was implemented, mapping camera images to control decisions. A convolutional neural network (CNN) with six convolution layers and two fully connected (FC) layers, sized 512 × 100 × 10, was initially trained off-chip using old scene data (specifically, dark scene data). The weights of the two FC layers were then transferred to the corresponding arrays on the memristor chip. Subsequently, improvement learning was performed on the chip for new scenes (bright scenes) by tuning the weights of the last FC layer. This demonstration showcased the computing-in-memory chip capability to adapt to different scenarios and enhance learning performance. In MNIST (mixed national institute of standards and technology) dataset task, the accuracy of the ordinary training is 90.7%, while the accuracy increases to 95.6% after training. Under the hybrid training framework, based on the influence of each layer of neural network on system accuracy, the weights are mapped to the chip, and then the improvement training is used to update the weights of critical layers while keeping unchanged of the non-critical layer weights, for further improving the system accuracy. With off-chip training on-chip improvement training, the accuracy values of the MNIST dataset and more complex CIFAR-10 dataset tasks can be revised from 95.6 to 96.9% and 82.0–94.4%, respectively.

## Methodology

The optimization approaches for devices, circuits, architectures and algorithms were comprehensively reviewed, and then cross-level co-simulation tools were developed to effectively address lack of EDA tool-chains and compilers related to memristors. The toolset comprises six essential components, including multi-scale atomic-level simulation framework at the bottom, Kinetic Monte Carlo (KMC) simulation platform, design and technology co-optimization tool, end-to-end simulator, hardware emulator and compiler. These tools provide comprehensive support for chip design, simulation, layout, and routing processes, thereby enhancing design efficiency and reliability of chip.

## Systems-on-Chips

With continuous development, the computing-in-memory chips have evolved from the initial prototype to wafer-level integrated systems and on-chip integrated Systems-on-Chips (SoCs). The latest reported C160 SoC is a RISC-V-based chip with large storage capacity. The computing performance can reach 100 TOPs, perform 2 trillion operations energy consumption per unit, and meet requirements of complex computing and storage. The computing-in-memory edge intelligence platforms have been successfully applied to support robust computational capabilities for AI and big data. These platforms facilitate efficient data processing and computation, thereby fueling the advancements in the edge intelligence applications.

## Conclusion and Perspective

In conclusion, the fully-integrated memristor chip for edge learning is of great significance in advancement of AI and big data. The adoption of “TiN/HfOx/TaOy/TiN” memristors stabilizes the conductance values during SET and RESET operations, resulting in a high programming success rate. By combining the emulation of dendritic structures in the neuromorphic system, the fully-integrated memristor chip enables efficient object recognition in noisy backgrounds while minimizing energy consumption. The utilization of crossbar-based memristor arrays facilitates efficient analog computation and in-memory computing, effectively addressing the IR drop issue with a 2T2R configuration. Furthermore, the integrated memristor chip incorporates the STELLAR architecture for on-chip learning, featuring a cycle-parallel conductance tuning scheme and integration of storage and computation. The algorithm employs a hybrid training framework, combining on-chip improvement training with off-chip training, to further enhance system accuracy. The methodology evaluates optimization approaches for devices, circuits, architectures, and algorithms, resulting in improved chip design efficiency and reliability. These advancements have led to the development of on-chip integrated SoCs with integrated storage and computing capability. To foster the advancement of computing-in-memory chips, it is crucial to promote interdisciplinary collaboration, enabling adaptation to diverse application scenarios and meeting the requirements of various stakeholders.

The computing-in-memory chip represents an innovative and interdisciplinary technology that extends beyond multiple domains, including mathematical theory, physical principles, semiconductor devices, nanoscale integration, circuit design, analog algorithms, software tools, compilers, and instruction sets. In mathematical theory, the Turing completeness must be proved to perform arbitrary computational tasks for computing-in-memory chip. In physical principle, it is desired to explore new operators for efficient computational operations. The high precision and consistency of semiconductor devices are required to ensure the stability and reliability of the chip. Integration scales of billions and even more must be with the aid of integrated nanoscale process to meet the high memory density of the chip. For design of device circuit, the data transmission and analog-to-digital conversion must be included. In analog algorithm, the efficient computational algorithms must be integrated on computing-in-memory chip. As for software tools, EDA tools and algorithm libraries must be supplied to design and develop the chip. Compilers need to achieve the unified compilation of storage and computation for the chip. In instruction set field, appropriate instruction set is imperative to be defined to support different computational tasks on the chip. Therefore, the computing-in-memory chip has the potential to transcend the traditional computing limitations and achieve more efficient and powerful computational capabilities.
